# Utilization and Complications of Central Venous Access Devices in Oncology Patients

**DOI:** 10.3390/curroncol28010039

**Published:** 2021-01-10

**Authors:** Narmeen Akhtar, Linda Lee

**Affiliations:** 1 Faculty of Applied Health Sciences, Brock University, St. Catharines, ON L2S 3A1, Canada; narmeen.akhtar98@gmail.com; 2 Department of Oncology, Niagara Health, St. Catharines, ON L2S 0A9, Canada; 3 Department of Oncology, McMaster University, Hamilton, ON L8V 5C2, Canada

**Keywords:** central venous access devices, peripherally inserted central catheter, implanted vascular access device, infections, venous thromboembolism, complications

## Abstract

Purpose: To describe how central venous access devices (CVADs) are utilized for ambulatory oncology patients and to evaluate the rate of complications. Method: Single institution retrospective study of oncology patients with CVADs who received systemic treatment at the Walker Family Cancer Centre (WFCC) between 1 January and 31 December 2018. Results: A total of 480 CVADS were placed in 305 patients, of which 408 (85%) were peripherally inserted central catheters (PICCs) and 72 (15%) were implanted vascular access devices (PORTs). The incidence of early and late complications was 9% and 24%, respectively. For the entire cohort, the rate of venous thromboembolism (VTE) was 16%, of which 9% were CVAD-related thrombosis (CRTs) and 7% were distant VTE. The CRT rates were similar for PICCs and PORTs (9% vs. 7%). A total of 6% of CVADs were complicated by infection (i.e., localized infections and bacteremia), with a total infection rate of 0.43 and 0.26 per 1000 indwelling days for PICCs and PORTs, respectively. The incidence of central line associated bloodstream infections (CLABSI) was greater for PICCs than PORTs, at a rate of 0.22 compared with 0.08 per 1000 indwelling days, respectively. The premature catheter removal rate was 26% for PICCs and 18% for PORTs. PORTs required more additional hospital visits. Conclusions: PICCs were utilized more frequently than PORTs and had a higher rate of premature removal. The rates of VTE and CRT were similar for both CVAD types. PORTs had a lower rate of infection per 1000 indwelling days. However, the management of PORT related complications required more visits to the hospital and oncology clinic.

## 1. Introduction

Intravenous cancer treatment, such as chemotherapy and immunotherapy, increase the survival and quality of life of patients with cancer. However, the administration of treatments repeatedly over a long period of time can be damaging to peripheral veins. Central venous access devices (CVADs) are used to prevent damage from repeated venipuncture and from vesicant or irritant drugs [1,2]. CVADs can also be used for supportive therapies such as medications, hydration, blood products, and total parental nutrition (TPN).

The two types of CVADs most commonly used in oncology patients are either peripherally inserted central catheters (PICCs) or implanted vascular access devices (PORTs). Guidelines recommend that a PICC should be inserted for patients expected to receive short duration treatment, while PORTs should be inserted if long term treatment is required [3,4]. CVADs are useful for administering systemic therapy in oncology patients, but are associated with potential complications. Short term complications include cardiac arrhythmia, bleeding, malpositioning, air embolism, or injury to vessels and nerves [5]. Long-term complications include catheter occlusion, venous thromboembolism (VTE), blood stream infection, migration, or mechanical dysfunction [6]. Early complications are generally related to insertion issues, while late complications are due to improper maintenance of the CVAD [7]. Infection and VTE are the two most serious complications of CVAD use that increase morbidity and lead to increased hospitalization and resource utilization. Both of these complications may be life-threatening or have serious sequelae such as halting cancer treatment, hospital admission, and the need for premature CVAD removal [5,6].

The purpose of this study is to examine the utilization of CVADs in oncology patients at our center, estimate the rate of complications, and evaluate the impact on hospital resource utilization.

## 2. Methods

This is a single center retrospective chart review of 305 oncology patients who received at least one intravenous systemic treatment at the Walker Family Cancer Centre (WFCC) and had a CVAD inserted between 1 January and 31 December 2018. The WFCC is a university-affiliated, community-based comprehensive cancer center in Southern Ontario where PICCs and PORTs are used as CVADs to administer systemic anti-cancer treatments. PICCs were inserted by experienced registered nurses (RNs) in the ambulatory oncology clinic, or by interventional radiologists in the hospital diagnostic imaging (DI) department. Single lumen 4Fr PowerPICCs™ (BD, Franklin Lakes, NJ, USA) were used in both locations. PowerPorts™ (BD, Franklin Lakes, NJ, USA) were inserted by interventional radiology and typically had a longer wait time. Standard protocols were used for PICC and PORT care at the WFCC, and these same protocols were provided to community nursing centers. This study was approved by the Hamilton Integrated Research Ethics Board.

### 2.1. Study Population

Eligible patients had a histologically confirmed diagnosis of malignancy, received at least one treatment with intravenous anti-cancer therapy (i.e., chemotherapy, targeted treatment, or immunotherapy), and had a CVAD inserted between 1 January to 31 December 2018. Patients were excluded if they had a CVAD that was used solely for supportive treatments such as transfusions or TPN.

### 2.2. Data Collection

Patients who had a CVAD inserted in 2018 were manually screened to see if they fit the inclusion criteria. Data were abstracted from their outpatient oncology and hospital electronic medical records, which were reviewed for events until 30 January 2020 in order to ensure there was a minimum of one year of follow up. The data collected included the diagnosis, year of diagnosis, year of treatment, intent of treatment (curative vs. palliative), Khorana Risk Score [8] to assess risk for venous thromboembolism (VTE) at time of initiation of systemic therapy (if available), type of CVAD, insertion and removal dates, insertion location, use of CVAD for bloodwork, and supportive intravenous therapies (i.e., hydration, antibiotics, medications, TPN, etc.). If an eligible patient had multiple CVADs inserted, information on all CVADs, including those inserted prior to and after the 2018 calendar year, as well as their complication events, were included.

### 2.3. Definition of Outcome Events

Events were considered only during the time that the CVAD was in-situ. Early complications were defined as events that occurred within seven days of insertion, and included bleeding, primary malpositioning, local infection, pain, and VTE. Late complications occurred more than seven days following the insertion date, and included catheter occlusion that did not resolve with thrombolytics, CRT, infection, migration, and mechanical dysfunction. VTE events were defined as symptomatic deep vein thrombosis (DVT) confirmed by Doppler ultrasound or pulmonary embolism (PE) evident on computed tomography (CT) imaging, whether symptomatic or incidental. CRTs were defined as symptomatic DVTs in the upper extremity ipsilateral to the CVAD, and were found to be in direct contact with the CVAD. Distant VTE events were defined as PE events or DVTs not in contact with the CVAD (i.e., DVT in the contralateral arm or in lower extremities). Infection events were identified as either localized infections (i.e., cellulitis at the insertion site or PORT pocket infection) or as laboratory confirmed bloodstream infections. Central line associated bloodstream infections (CLABSIs) were defined according to the Center for Disease Control (CDC) classification of bloodstream infections [9]. Infections were classified as a CLABSI if there was a laboratory confirmed bloodstream infection with an eligible pathogen in the absence of another source of infection, with the exclusion of cases that met CDC criteria for mucosal barrier infection (i.e., typical pathogen in the presence of neutropenia or significant diarrhea). Pharmacy records were reviewed to collect the use of antibiotics and systemic anticoagulation. Premature CVAD removal was defined as a removal due to complication events, patient request for an alternative CVAD type, or removal of a CVAD within 28 days of insertion of a new CVAD while continuing on the same systemic treatment. CVAD replacement was defined as exchange of the same CVAD type within seven days. The CVAD conversion rate included any change from one type of CVAD to an alternative CVAD type. Data on extra hospital, emergency room (ER), or oncology clinic visits outside of scheduled appointments to see their oncologist or to receive systemic treatment were also collected.

### 2.4. Statistical Analysis

Descriptive statistics were used to define the study population and CVAD utilization. Frequencies were used to define the characteristics of the CVAD and to compare the rates of complications, VTEs, infections, replacement, and premature removal. The rate of infection was also calculated based on the number per 1000 indwelling catheter days. Resource utilization was estimated by determining the number of extra visits to the outpatient oncology clinic, ER, or need for inpatient admission due to CVAD-related problems.

## 3. Results

The study included 305 patients with a median age of 66 (range, 20–90), as shown in Table 1. The majority of patients were female (58) and had early stage or curable cancers (57%). Within this population, the most common diagnosis was gastrointestinal cancer (33%). The most common systemic treatments in this population included doxorubicin (25%), 5-fluorouracil (23%), cisplatin (20%), and carboplatin (14%). Of the 305 patients, 7% had a history of VTE (5% DVT, 2% PE, and none were CRTs), 11% were on anticoagulants, 20% were taking antiplatelet agents, and 17% were diabetic. The majority of patients had an intermediate risk Khorana score (49%). In this study, patients had between one to six CVADs inserted, corresponding to an average of 1.6 CVADs per patient. The majority of patients had one CVAD (62%), but 25% had two, and 13% had three or more CVADs (Table 1). Only nine patients (3%) received a PORT as their first and only CVAD device.

The characteristics from 480 CVADs are shown in Table 2. The majority of CVADs inserted were PICCs (85%). Of the 480 CVADs, 72% were inserted by RNs in the outpatient oncology clinic. The majority of CVADs were used solely for the administration of anticancer treatment. Only a minority of CVADs had secondary use for hydration or transfusion (12%), intravenous supportive care medications (3%), both medication and hydration (3%), and TPN (1%). CVADs had an early complication rate of 9%, with bleeding as the most common event (3%). The late complication rate was 24%, with migration as the most common long-term complication (10%). A total of 78 VTEs (16%) occurred, including 42 (9%) CRTs and 36 (7%) distant VTEs. Only four VTEs were recorded in patients on anticoagulation. There were a total of 28 (6%) infections, including 10 (2%) localized infections and 13 (3%) CLABSIs, and five (1%) cases of bacteremia that were unrelated to the CVAD.

Table 3 describes the outcomes of the 408 PICCs and 72 PORTs included in this study. The median lifespan of a PICC was 99 days (range 1–891) compared with 344 days (range 9–1050) for a PORT. For PICCs, the VTE incidence was 17%, 9% of which were CRTs and 8% were distant VTE. For PORTs, the VTE incidence was 14%, of which 7% were CRT and 7% were distant VTE.

The incidence of infection in patients with PICCs and PORTs was 5% and 10%, respectively (Table 3). For both PICCs and PORTs, the incidence of CLABSI in each was equivalent at 3%. However, when accounting for the indwelling time, the rate of total infections per 1000 indwelling days was higher for PICCs than PORTs, at 0.43 and 0.26, respectively. The rate of CLABSIs was also higher for PICCs than PORTs, at 0.22 compared with 0.08 per 1000 indwelling days.

Premature catheter removal due to complications or request for a new CVAD occurred in 26% of patients with a PICC and in 18% of patients with a PORT (Table 3). The most common reason for premature removal was malposition or mechanical dysfunction, with a rate of 15% in PICCs and 11% in PORTs. A total of 16% of PICCs required replacement with another PICC and 10% of PORTs were replaced with another PORT. The rate of conversion to an alternative CVAD was 10% for PICCs converting to a PORT and 3% for PORTs converting to a PICC. The two patients who requested a PICC following a PORT both had PORT removal as a result of infection. A total of 54 patients (18%) had a PICC placed initially and were subsequently converted to a PORT. One request for conversion to a PORT was unsuccessful as the patient had situs inversus.

Extra hospital visits to the ambulatory oncology clinic, ER, and to the hospital for inpatient admission are shown in Table 3. For those with PICCs, 33% of devices required an extra visit to the oncology clinic, 10% required assessment in ER, and 5% were admitted to hospital. For those with PORTs, 40% of devices required an extra oncology clinic assessment, 11% required an ER visit, and 11% required inpatient admission. Of the 21 patients with PICCs requiring hospitalization, ten had CLABSIs, six had an unrelated infection, four had PE, and one developed an infected CRT. For the six patients with PORTs that required inpatient admission, three had CLABSIs, two had unrelated bacteremia, and one developed multiple distant DVTs. Therefore, about half of inpatient admissions were due to CVAD complications for both CVAD types.

## 4. Discussion

Venous access is a critical issue for oncology patients, as most systemic treatments are administered intravenously. Practice guidelines recommend the insertion of central venous access devices (CVADs) for the administration of any vesicant chemotherapy and for those requiring continuous infusions [3,4]. In a single year, our department required 480 CVADs to be inserted into 305 patients undergoing systemic treatment, which underscores the importance of this issue for oncology patients and highlights the significant resources required to insert and maintain CVADs.

While the majority of patients required only one CVAD, 117 (38%) patients had two or more CVADs and only nine (3%) of patients received a PORT as their first and only CVAD. PICCs had a shorter median lifespan and higher rates of replacement and premature removal. This finding may have partially been due to the greater access to PICC resources in DI and in the ambulatory oncology clinic, such that the wait times for insertion and exchange were shorter. PICCs are typically inserted if systemic treatment is required within two weeks, and if removal is required, can be exchanged on the same day or be replaced within a week. Conversely, PORTs are only inserted in the DI department and are typically associated with a longer wait time of up to three weeks, which may have impacted the initial CVAD selection, as demonstrated in a previous survey of Canadian health care professionals [1].

The development of VTE is a significant issue as it contributes to morbidity. Patients are usually symptomatic at presentation and treatment with systemic anticoagulation is required. For patients with curative disease, anticoagulation can usually be discontinued following CVAD removal and a minimum duration of treatment. However, the need for anticoagulation is often indefinite in patients with metastatic disease who are on continuous treatment and require long term use of a CVAD. Our study found an overall VTE rate of 16% (17% in PICCs and 14% in PORTs), with a nearly equal distribution between CRTs and distant VTEs. A previous retrospective study of PICCs found a higher VTE rate of 26%, but similarly found equivalent CRT and distant VTE rates of 13% for each [10]. However, this previous study also found that use of fluoropyramidine was associated with a 10-fold increased VTE risk, so the high rate of VTE found in this study was likely related to underlying patient and treatment factors, as 60% of the patients had metastatic disease, 55% had gastrointestinal cancers, and 80% received fluoropyramidine containing chemotherapy. The VTE rates for PORTs in our study were comparable to two previous prospective studies, in which the VTE rates at 12 months were 13.1% [11] and 15.3% [12]. In both of these studies, the distribution of cancer types and the proportion of metastatic patients were similar to our study population. One of these studies used the same definitions for CRT and distant VTE as our study and found a higher rate of distant VTE than CRT (9.6% vs. 3.8%), but thromboprophylaxis was administered to some patients following PORT insertion, which may have contributed to the low CRT rate [11]. In a prospective Canadian study of PORTs, the CRT was substantially lower at 1.3%, but the PORTs utilized contained a novel anti-thrombogenic polymer and were maintained exclusively by a specialized team of RNs [13]. While our study described similar CRT rates for PICCs and PORTs (9 vs. 7%), it is notable that other studies that directly compared PICCs and PORTs demonstrated that PORTs were associated with a reduced risk of CRT [6,14,15,16]. The majority of patients in our study received their PORT following the insertion/removal of another CVAD, and were likely more advanced in their treatment course, which may have impacted the VTE and CRT rates for our PORT cohort.

Cancer patients on immunosuppressive therapy are at higher risk of infection and the presence of a CVAD, which can become colonized with bacteria, further increases this risk. Development of a CLABSI results in morbidity for patients, as management usually involves inpatient or intensive care admission, use of intravenous administration of antibiotics, and removal of the CVAD. Lebeaux et al. reported that amongst 72 oncology patients with CLABSIs, 18% presented with severe sepsis or shock, and after 12 weeks, 30% had to discontinue chemotherapy and 46% died [17]. The CLABSI rate of 0.22 per 1000 indwelling days in PICCs in our study was similar to reported rates in the existing literature, which varied from 0.12 to 0.98 per 1000 indwelling days [7,18,19,20]. However, our finding of 0.08 CLABSIs per 1000 indwelling days in PORTs was lower than in other studies, in which the rates ranged between 0.21 to 0.76 infections per 1000 indwelling days [21,22]. This may have been due to our strict definition of CLABI according to CDC classification, which excludes infections attributed to mucosal barrier injury [9] in the context of diarrhea or neutropenia, both of which are common in cancer patients on treatment. Our findings also contrasted with a meta-analysis that demonstrated that PICCs are associated with a lower risk of CLABSI in the outpatient setting, although this study was not conducted exclusively in oncology patients [23]. In our patient population, PICCs were maintained exclusively by community-based RNs. PORTs were maintained by specialized RNs within our oncology clinic while patients were receiving systemic treatment and, subsequently, by community-based RNs once treatment was completed. Despite using the same protocols for care, it is possible that this difference contributed to the higher infections rates in patients with PICCs. Proper CVAD maintenance is important for preventing infections, and in a prospective Canadian study of 389 patients with PORTs maintained by a specialized RN team, there were no CLABSIs observed within a 12-month period [13].

Although PORTs were associated with a greater number of extra hospital visits, PICC line complications were associated with a higher replacement and/or removal rate. A total of 28% of PICCs were removed prematurely compared with only 18% of PORTs. Our PICC removal rate due to complications was higher than that previously reported in the literature of 11–15% [18,19], but the rate of PORT removal was similar to a previous study [24]. One advantage of PICCs is that they are easily removed, so this intervention may have prevented further complications such as worsening infection. A previous study showed that preventative measures to decrease the number of emergency visits included improved higher patient education about their CVAD, frequent flushing of the device, and frequent dressing changes [5].

The main limitation of our study is that it is a descriptive study, and while we examined events for PICCs and for PORTs, a formal statistical comparison was not possible. The majority of our patients had a PICC prior to a PORT, such that the two cohorts overlapped. Because of the wait time for PORTs, PICCs were generally selected for patients with curative disease who were expected to be on short-term treatment and who needed to start treatment within a two-week timeframe. Our cancer center rigorously follows the Registered Nurses’ Association of Ontario best practice guidelines [4], such that all patients receiving vesicants are strongly encouraged to have a CVAD inserted, and all patients receiving irritants are offered a CVAD prior to starting systemic treatment. Thus, we likely have a larger number of patients with CVADs and greater CVAD utilization in cancer types requiring anthracyclines, such as breast and hematologic cancers. As such, the single-center design of this study limits the generalizability of our results, as VTE rates are associated with cancer site and type of chemotherapy [8,10,11,12,19], and infection rates are associated with cancer type and curative versus palliative treatment [7,21,22].

Among 305 patients, only nine had a PORT inserted as their first and only CVAD, while 54 patients were converted from a PICC to a PORT. Conversion from one CVAD to another suggests that there is room to optimize up front CVAD selection and reduce resource utilization necessary for inserting two types of devices. Current guidelines report insufficient evidence to recommend one type of CVAD over another [5,25]. A survey of oncologists reveals that CVAD selection is driven by patient preference, access to CVAD insertion services, and individual patient risk factors [1]. A history of deep-vein thrombosis, age, use of the subclavian vein for CVAD insertion, comorbidities such as diabetes, and use of the CVAD for supportive therapy have been shown to increase the risk of infections and VTE in oncology patients with a PICC [7]. A randomized study of PICCs compared with PORTs found that the rate of PORT complications was less [6]. However, PORT complications are associated with serious morbidity, including hospitalization for sepsis, hematogenous spread of infection, and cessation of anti-cancer therapy [17,26]. Peripheral IVs (PIV) are another possibility for those who are receiving short term treatment. As PIVs have a higher risk of chemotherapy extravasation, evaluation of patients risk factors such as vein fragility and the type of chemotherapy (i.e., irritant or vesicant) may allow some patients to completely avoid the use of a CVAD [1,27].

## 5. Conclusions

Our study describes the utilization and rate of complications for PICCs and PORTs within an outpatient oncology population. We found complications were common and occurred with one third of all CVADs. The risk of complications may outweigh the potential benefits of a CVAD in some patients, particularly in situations where treatments are short-term and do not contain either a vesicant or continuous infusion. Proper vein assessment may mitigate the need for a CVAD in some patients. In patients where a CVAD is necessary, an optimal device should be selected up front based on patient preference, expected treatment duration, and consideration of potential complications, which should be discussed with patients at the time of CVAD selection. We found that the rates of VTE and CRT were similar for PICCs and PORTs, but PORTs had a lower rate of CLABSI per 1000 indwelling days, a longer median lifespan, and lower rates of replacement or premature catheter removal. If there is equal access to either PICCs or PORTs, our study supports that PORTs should be recommended for palliative patients who usually require long term treatments and have a higher risk of infection. Increased utilization of PORTs for these patients would decrease the resources needed for the insertion and replacement of multiple CVADs, but greater hospital resources may be required for their maintenance.

## Figures and Tables

**Table 1:** Patient characteristics (*n* = 305).

Characteristic	*n*	%
**Age at CVAD placement**		
Median	66	
Range	20–90	
**Gender**		
Female	176	58
Male	129	42
**Type of cancer**		
Breast	68	22
GI	102	33
Gyne	18	6
GU	7	2
Lung	48	16
Hematology	53	17
Skin	2	1
Other	7	2
**Stage**		
Early Stage	174	57
Advanced/metastatic	131	43
Type of systemic treatment *		
Cisplatin containing	62	20
Carboplatin containing	44	14
Doxorubicin containing	76	25
5-Fluorouracil containing	71	23
Anti-angiogenic agent containing	22	7
**History**		
Diabetes	53	17
Previous VTE	22	7
**Medications**		
Anti-platelet agents, total	60	20
Anti-coagulants, total	35	11
LMWH	8	3
NOACs	21	7
Warfarin	6	2
**Khorana Risk Score**		
0	93	31
1–2	150	49
3 or greater	25	8
Unknown	37	12
**Number of CVADs**		
1	188	62
2	76	25
3 or more	41	13

* Systemic treatments may have included a combination of more than one of the listed drug types (i.e., if a patient had 5-fluorouracil and cisplatin, it would be counted once in each category). CVAD—central venous access device; GI—gastrointestinal; Gyne—gynecological; GU—genitourinary; VTE—venous thromboembolism; LWMH—low molecular weight heparin; NOAC—novel oral anticoagulants (i.e., apixaban, dabigatran, rivaroxaban, and edoxaban).

**Table 2:** CVAD characteristics (*n* = 480).

CVAD Characteristic	*n*	%
**Type of device**		
PICC	408	85
PORT	72	15
**Side of placement**		
Left	114	24
Right	362	75
Not recorded	4	1
**Location of insertion**		
Oncology clinic by nurse	345	72
DI by radiologist	133	28
External hospital	2	0
**Use for intravenous supportive care**		
Not used	389	81
Hydration or transfusion	59	12
Medication only	13	3
Both hydration/transfusion and medication	14	3
TPN	6	1
Early complications (within 7 days), total	43	9
Bleeding	17	3
Malposition	10	2
Pain	8	1
CRT	3	1
Local site infection	5	1
Late complications, total	116	24
CRT	39	8
Infection *	17	3
Migration	47	10
Catheter occlusion	8	2
Mechanical dysfunction	5	1
VTE rate, total	78	16
CRT	42	9
Distant VTE	36	7
Infection rate, total	28	6
Localized around CVAD	10	2
CLABSI	13	3
Bacteremia unrelated to CVAD	5	1

* One patient had an infected CRT, which was categorized as a CRT event in late complications, but was considered separately as a VTE and an infection event in VTE and infection rates. CVAD—central venous access device; PICC—peripherally inserted intravenous catheter; PORT—implanted vascular access device; DI—diagnostic imaging; TPN—total parental nutrition; CRT—catheter related thrombosis; VTE—venous thromboembolism; CLABSI—central line associated bloodstream infection.

**Table 3:** Description of outcomes by CVAD type.

Characteristic	PICC (*n* = 408)		Port (*n* = 72)	
	*n*	%	*n*	%
**CVAD lifespan, days**				
Median	99		344	
Range	1–891		8–1050	
**Venous thrombosis event, total**	68	17	10	14
CRT	37	9	5	7
Distant VTE	31	8	5	7
PE	20	5	1	1
DVT	11	3	4	6
**Infection rate, total**	21	5	7	10
Localized	7	2	3	4
CLABSI	11	3	2	3
Bacteremia unrelated to CVAD	3	<1	2	3
**Infection per 1000 indwelling days, total**	0.43		0.26	
CLABSI per 1000 indwelling days	0.22		0.08	
**Premature catheter removal, total**	108	26	13	18
Due to infection	13	3	5	7
Due to malposition/dysfunction	60	15	8	11
Request for alternative CVAD	35	9	-	-
Replacement rate				
PICC exchange	65	16	-	-
PORT exchange	-	-	7	10
Conversion rate				
PICC to PORT	39	10	-	-
PORT to PICC	-	-	2	3
Extra hospital visits				
Oncology clinic	135	33	29	40
Emergency department	42	10	9	12
Inpatient admission	21	5	6	8

CVAD—central venous access device; CRT—catheter related thrombosis; VTE—venous thromboembolism; PE—pulmonary embolism; DVT—deep vein thrombosis; CLABSI—central line associated bloodstream infection; PICC—peripherally inserted central catheter; PORT—implanted vascular access device.

## Data Availability

The data presented in this study is available on request from the corresponding author (L.L.). The data is not publicly available in order to protect patient confidentiality.
